# The guiding effect of local field potential during deep brain stimulation surgery for programming in Parkinson's disease patients

**DOI:** 10.1111/cns.14501

**Published:** 2023-10-13

**Authors:** Wenwen Dong, Chang Qiu, Lei Chang, Jian Sun, Jiuqi Yan, Bei Luo, Yue Lu, Weiguo Liu, Li Zhang, Wenbin Zhang

**Affiliations:** ^1^ Department of Functional Neurosurgery The Affiliated Brain Hospital of Nanjing Medical University Nanjing China; ^2^ Department of Neurology The Affiliated Brain Hospital of Nanjing Medical University Nanjing China; ^3^ Department of geriatric medicine The Affiliated Brain Hospital of Nanjing Medical University Nanjing China

**Keywords:** deep brain stimulation, local field potential, parkinson's disease, power spectral density, programming

## Abstract

**Background:**

Parkinson's disease (PD) patients undergoing deep brain stimulation (DBS) surgery require subsequent programming, which is complex and cumbersome. The local field potential (LFP) in the deep brain is associated with motor symptom improvement. The current study aimed to identify LFP biomarkers correlated with improved motor symptoms in PD patients after DBS and verify their guiding role in postoperative programming.

**Methods:**

Initially, the study included 36 PD patients undergoing DBS surgery. Temporary external electrical stimulation was performed during electrode implantation, and LFP signals around the electrode contacts were collected before and after stimulation. The stimulating contact at 6 months of programming was regarded as the optimal and effective stimulating contact. The LFP signal of this contact during surgery was analyzed to identify potential LFP biomarkers. Next, we randomly assigned another 30 PD patients who had undergone DBS to physician empirical programming and LFP biomarker‐guided programming groups and compared the outcomes.

**Results:**

In the first part of the study, LFP signals of electrode contacts changed after electrical stimulation. Electrical stimulation reduced gamma energy and the beta/alpha oscillation ratio. The different programming method groups were compared, indicating the superiority of beta/alpha oscillations ratio‐guided programming over physician experience programming for patients' improvement rate (IR) of UPDRS‐III. There were no significant differences in the IR of UPDRS‐III, post‐LED, IR‐PDQ39, number of programmings, and the contact change rate between the gamma oscillations‐guided programming and empirical programming groups.

**Conclusion:**

Overall, the findings reveal that gamma oscillations and the beta/alpha oscillations ratio are potential biomarkers for programming in PD patients after DBS. Instead of relying solely on spike action potential signals from single neurons, LFP biomarkers can provide the appropriate depth for electrode placement.

## Graphical abstract

Gamma oscillations and the beta/alpha oscillations ratio are potential biomarkers for programming after DBS in PD patients.

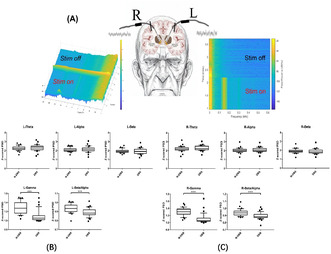

## 1 INTRODUCTION

Parkinson's disease (PD) is the second most common neurodegenerative disease globally, and its prevalence increases with age.^1^, ^2^ The clinical manifestations of PD primarily include motor symptoms, such as bradykinesia, resting tremor, and limb stiffness, and non‐motor symptoms, like hyposmia, sleep disorders, and cognitive impairment.^2^ In the early stages of the disease, oral anti‐PD drugs can help improve symptoms. However, PD patients often face challenges such as increased drug dosage, shortened drug effect time, the “on–off” fluctuations phenomenon, and dyskinesia with disease progression.^3^ After strict preoperative evaluation, some advanced PD patients undergo deep brain stimulation (DBS) surgery to improve motor symptoms. DBS surgery involves the implantation of one or more electrodes into a specific area of the patient's brain. The electrodes are connected to an implantable pulse generator using an extended electric wire. The pulse generator sends electrical pulses to the brain tissue, and the surgeon intervenes in the abnormal neural circuits by adjusting the pulse parameters to improve PD symptoms.^4^, ^5^

After implantation, adjustments are made to the electrode stimulation parameters, such as the voltage, current, frequency, and pulse width.^6^ DBS programming involves changing the stimulation parameters to improve symptoms and reduce side effects.^7^ Experienced physicians generally perform DBS programming. The implanted electrodes have four stimulating contacts at the end. During the programming process, achieving individual electrical or combined stimulation of each contact is necessary to determine the optimal stimulating contact, which can be time‐consuming.^8^ The emergence of directional electrodes,^9^ increased stimulating electrode contacts,^10^ and increased stimulation modes^11^ have further elevated the programming difficulty and associated workload for doctors. As a result, there is an urgent need to identify biomarkers to improve programming efficiency. Recent studies have reported that PD symptoms may be associated with the local field potential (LFP) of the basal ganglia.^12^, ^13^, ^14^, ^15^, ^16^, ^17^, ^18^ Therefore, the primary purpose of this study was to identify potential biomarkers by analyzing the LFP signals of electrode contacts and to guide programming after DBS using LFP biomarkers.

## 2 MATERIALS AND METHODS

This study was approved by the Human Participants Ethics Committee of the Affiliated Brain Hospital of Nanjing Medical University. All the patients or their family members provided written informed consent for study participation.

### 2.1 Study design and patient groups

From January 2016 to August 2022, 108 patients with primary PD were recruited from the Department of Functional Neurosurgery, Nanjing Brain Hospital, Affiliated to Nanjing Medical University. The inclusion criteria were (1) a definite diagnosis of idiopathic Parkinson's disease, (2) no dementia or major mental illness, (3) parkinsonian motor symptoms or dyskinesias limiting activities of daily living, (4) persistence of symptoms despite optimal medical therapy, (5) no surgical contraindications, (i.e., can remain calm and cooperative during surgery), and (6) willingness and ability to make subsequent visits.^19^, ^20^

Due to data quality and loss of follow‐up, 66 patients were ultimately included in this study. Of these, 36 were in the LFP biomarkers group (LBG), and 30 were in the programmed validation group (PVG).

#### 2.1.1 LBG group

**FIGURE 1:**
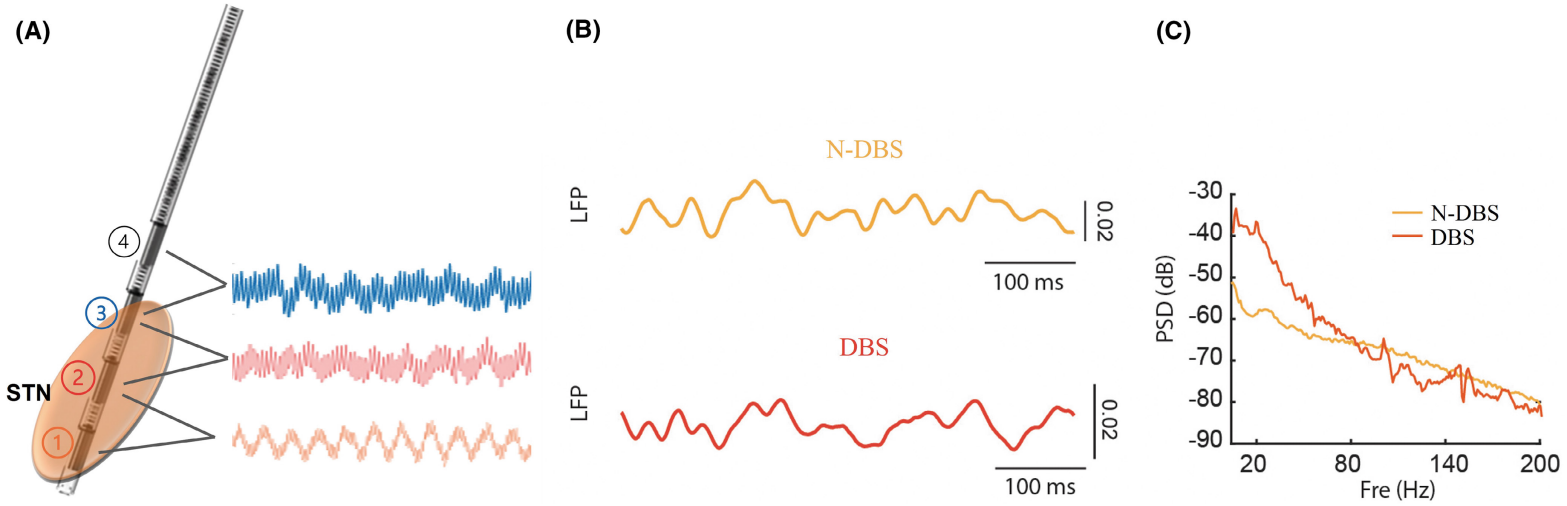
(A) Schematic diagram of the electrodes implanted into the subthalamic nucleus; ①②③④ represent the four contacts of the electrode. The LFP signals of the electrode contacts can be collected through the adjacent contacts. (B) Refers to the part of the acquired LFP signal, N‐DBS represents the field potential signal acquired without stimulation, and DBS represents the field potential signal with stimulation. (C) The collected field potential signals were Fourier transformed into power spectra, with the abscissa Fre representing the frequency and the ordinate PSD representing the power spectrum.

Electrodes were implanted under local anesthesia, and temporary external electrical stimulation (parameters: 1–4+, 2.5 V, 60 μs, 150 HZ) was then applied. The LFP signals between the contacts were collected before and after electrical stimulation (Figure 1A,B), and then we analyzed the power spectrum of LFP by Fourier transform. One month after DBS surgery, electrical stimulation was performed in the monopolar stimulation mode. At 6‐month follow‐up, the stimulation parameters and clinical characteristics were collected. The stimulating contact during this time was regarded as the optimal effective stimulating contact. According to the stimulating contact, the intraoperative LFP signal of the contact was retrospectively analyzed. Potential LFP biomarkers were obtained by analyzing the LFP signal changes before and after temporary external stimulation.

#### 2.1.2 PVG

This group underwent the same surgical procedure as the LBG group. Electrodes were implanted using a neuro‐smart electrophysiological device (Alpha Omega Engineering) and HaGuide Brain Map (Alpha Omega Engineering). The LFP signals at each contact were collected and analyzed. Then, patients were randomized to receive empirical programming or LFP biomarker‐guided programming.

The clinical data of all the included PD patients were collected (Table 1), such as gender, age, disease course, preoperative daily levodopa equivalent dose (pre‐LED), Unified Parkinson's Disease Rating Scale part III score in the preoperative medication off‐period (pre‐UPDRS III), Hamilton Anxiety Scale (HAMA) score, Hamilton Depression Scale (HAMD) score, and the Montreal Cognitive Assessment (MOCA).

**TABLE 1:** Demographic and clinical characteristics of the included patients.

	LBG (*n* = 36)	PVG (*n* = 30)	*p*‐value
Gender (female/male)	22/14	9/21	0.450
Age (years)	55.5 ± 7.84	60.3 ± 6.82	0.011
CD (years)	8 (7,11.75)	8 (6,11.25)	0.916
Pre‐LED (mg)	622.83 ± 117.76	707.53 ± 120.35	0.005
Pre‐UPDRS III	64.08 ± 17.35	51.13 ± 11.62	0.001
HAMA	5.36 ± 3.23	9.47 ± 3.69	<0.001
HAMD	9 (6,13.75)	13 (5,16.25)	0.530
MOCA	25.81 ± 2.51	26 (23.75,29.25)	0.835

*Note*: Data are presented as the mean ± SD or median (25th–75th percentile).

Abbreviations: CD, course of the disease; HAMA, Hamilton Anxiety Scale; HAMD, Hamilton Depression Scale; MOCA, Montreal Cognitive Assessment; Pre‐LED, preoperative daily levodopa equivalent dose; pre‐UPDRS III, Unified Parkinson's Disease Rating Scale part III of the preoperative medication off period.

### 2.2 DBS surgical procedure

Before surgery, all the patients underwent 3.0 T magnetic resonance imaging (MRI) scanning involving T1, T2, and SWI sequences. The Leksell SurgiPlan Planning System (Elakta) helped plan the preoperative electrode path. Electrodes were implanted using the Leksell Stereotactic System (Elakta). The target location for implantation was the subthalamic nucleus (STN). The intracranial stimulation electrodes were all model L301 (PINS). Before electrode implantation, single neuron spike action potential signals were recorded using a microelectrode recording (MER) needle (Alpha Omega Engineering). When the MER needle tip first entered the STN, neuronal burst electrical signals were recorded. The MER needle was further pushed down until the neuronal electrical signals disappeared; this indicated that the needle tip exited the STN, and this position was determined as the lowest point of electrode implantation. Afterward, the implantable pulse generator (IPG) was implanted under general anesthesia.

### 2.3 LFP recording

For the LBG group, after implantation of intracranial stimulating electrodes into the STN, LFP signals around adjacent contacts of stimulating electrodes were collected using the EEG 100C module of the MP‐150 physiological multichannel device (BIOPAC Systems, Inc). LFPs were generally recorded for 120 s after the signal was stable. Patients were in an awake resting state during DBS surgery. A head computed tomography (CT) scan was performed within 24 h post‐surgery. The postoperative CT and preoperative 3.0 T MRI were fused using the Leksell Surgical Planning System to confirm electrode implantation in the STN.

For postoperative programming, the IPG was turned on 1 month after DBS surgery in the monopolar stimulation mode. The electrical stimulation parameters were adjusted several times for each patient's motor symptoms. Generally, motor symptoms in PD patients are controlled and remain stable 6 months after IPG start‐up. The following data were collected for patient programming and related clinical characteristics: improvement rate (IR) of the Unified Parkinson's Disease Rating Scale part III (UPDRS‐III) during medication‐off (med‐off), postoperative daily levodopa equivalent dose (post‐LED), IR of the Parkinson's Disease Questionnaire (PDQ‐39), number of programmings, and ratio of the number of changes within the patient's stimulus contacts during the follow‐up programmed control to that of programmed controls (contact change rate).

### 2.4 Data processing and analysis

The stimulation contact after 6 months of programming was regarded as the optimal stimulation contact. The LFP signals recorded by the corresponding contact channels during surgery were selected. For data extraction, LFP signals recorded using an MP‐150 physiological multichannel instrument (sampling rate 2000HZ) were stored in an acq structure and then transformed into a mat structure for further analysis.

For the power spectrum density (PSD) analysis, signal processing was performed using custom scripts in MATLAB (version 2018a, MathWorks). We used a notch filter to remove artifacts at 25 Hz, 50 Hz, 75 Hz, 100 Hz, 125 Hz, and 150 Hz. The PSD estimate was calculated using Welch's method (1‐s sliding window, 25% overlap). The averaged values were calculated in different bands (delta band: 5–8 Hz, alpha: 8–12 Hz, beta band: 12–30 Hz, gamma band: 50–200 Hz) for statistical comparisons.^21^, ^22^

### 2.5 Statistical analysis

Statistical analysis was performed using GraphPad Prism 9 (GraphPad Software). Shapiro–Wilk's test helped confirm the normality variance assumptions for all the variables. Data meeting a normal distribution are expressed as the mean ± standard deviation (x ± sd), while those not satisfying a normal distribution are defined as the median (25th–75th percentile). Two‐sample *t*‐tests or analysis of variance (ANOVA) were used for comparisons between groups for data satisfying a normal distribution. In contrast, the Kruskal‐Wallis test compared groups for data not satisfying a normal distribution. Levene's test confirmed the homogeneity assumptions. One‐way ANOVA was used for comparisons between groups with equal variance, while Welch's ANOVA was used for comparisons between groups with unequal variance. For all the tests, the statistical significance level was *p* < 0.05.

## 3 RESULTS

### 3.1 Patient demographic and clinical characteristics

As shown in Table 1, the LBG and PVG groups did not differ significantly in terms of gender, CD, HAMD, or MOCA scores. However, there were significant group differences in age, pre‐LED, pre‐UPDRS III, and HAMA scores during the drug‐off period. Patients in the LBG group were slightly older, took more oral medications, and were relatively more anxious but had lower UPDRS III scores during the preoperative drug‐off period than the PVG group.

### 3.2 Identification of potential LFP biomarkers

**FIGURE 2:**
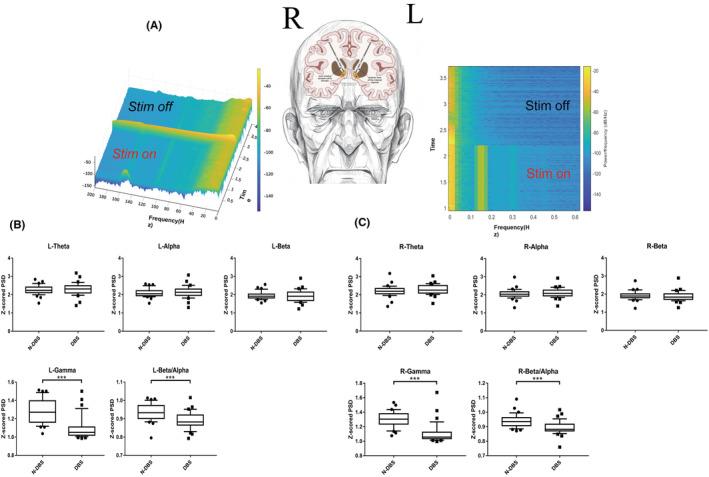
(A) Time–frequency plots of field potentials on the left and right sides of PD patients. Stim on represents stimulus on, stim off represents stimulus off, and brighter colors represent higher energy. (B) LFP energy in the left deep brain. (C) LFP energy in the right deep brain. *p < 0.05, ***p < 0.001.

As shown in Figure 1A, we obtained LFP signals around the stimulating contacts of the electrodes through the deep brain electrodes. We obtained two kinds of LFP signals when stimulation was switched on and off by connecting electrode leads and giving temporary electrical stimulation (Figure 1B). Fourier transformation helped provide the PSD of LFP. Figure 1C shows that the PSD of LFP without electrical stimulation (N‐DBS) and with electrical stimulation (DBS) may differ between 0–80 Hz. This finding suggests that electrical stimulation can change the LFP signal in the deep brain. Next, we separately analyzed the LFP signals of the left and right electrodes, as shown in the time‐frequency diagram of the left electrode (the external stimulus wave of 150 HZ was not filtered out) and the field potential of the right electrode in Figure 2A. The LPF energy in the stimulation on (stim on) period was about 50–80 HZ lower than in the stimulation off (stim off) period. Following the previous studies, we subdivided the field potential oscillations into delta (5–8 Hz), alpha (8–12 Hz), beta (12–30 Hz), and gamma (50–200 Hz) bands.^21^, ^22^ There were significant differences in gamma oscillations before and after electrical stimulation. However, beta oscillations, commonly associated with PD, did not show significant differences before and after stimulation. For further analysis, we compared the beta/alpha oscillation ratios. Interestingly, there was a statistically significant (*p* < 0.001) difference in the beta/alpha oscillation ratios before and after stimulation.

### 3.3 Validation of LFP biomarkers

**FIGURE 3:**
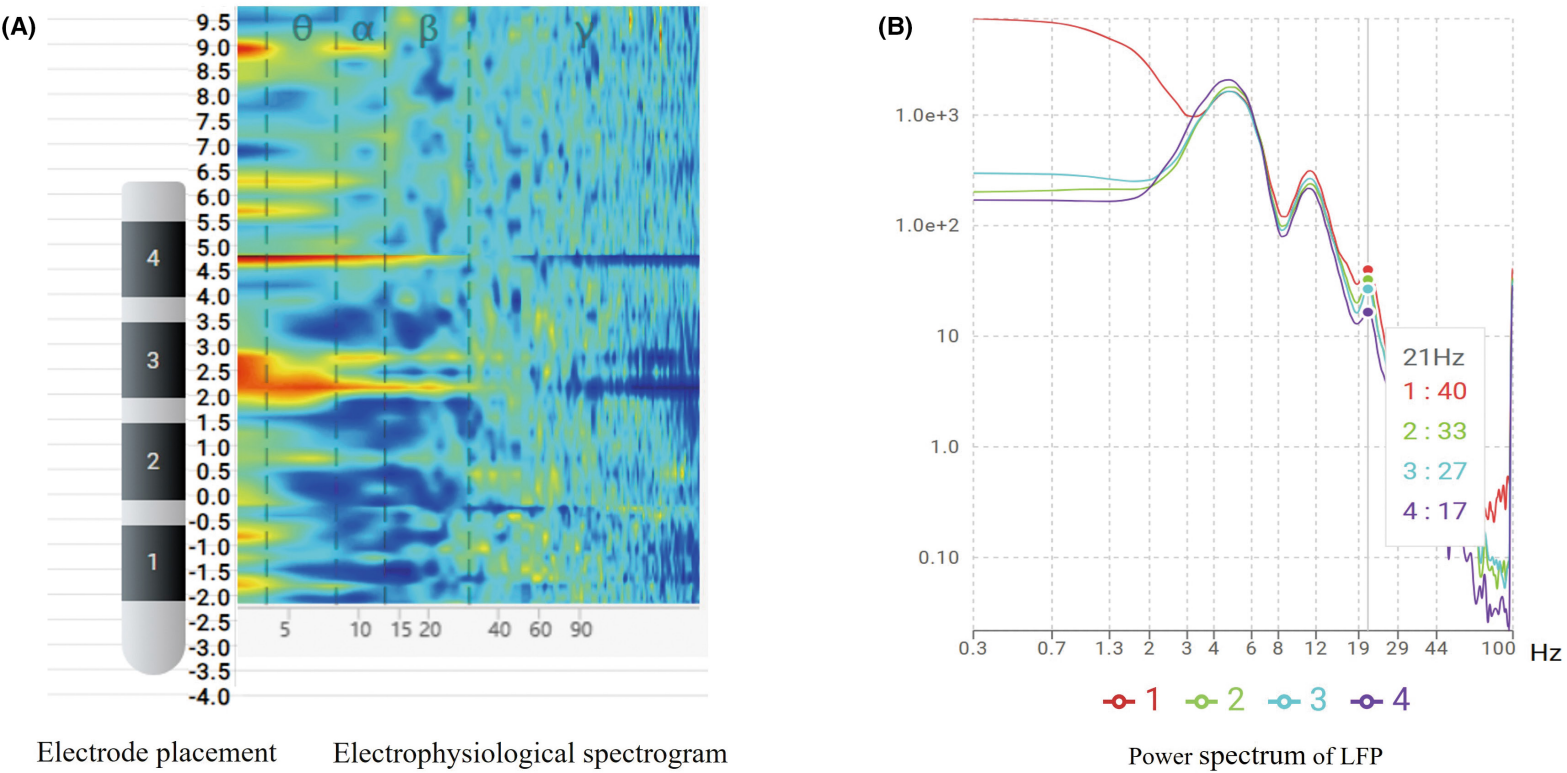
(A) The field potential spectrogram at the electrode implantation site. The depth of electrode implantation is 2 mm below the target. A brighter color of the spectrogram represents higher energy. (B) The power spectrum energy map of the LFP at each contact is shown.

Before implantation, the depth of the electrode must be confirmed following the neuronal electrical signals using the MER needle. Then, the LFP signals at different depths were obtained using the MER needle. Figure 3A shows that the depth of the electrode implantation site is 2 mm below the target site (the target site is 0); the brighter color of the spectral energy map at different depths indicates higher energy. After the electrodes were implanted and connected with the external lead, the field potential power spectrum of each contact was obtained using the Haguide software (Figure 3B). The postoperative programmed stimulation contacts were selected according to the gamma oscillation and beta/alpha oscillation energy values.

**FIGURE 4:**
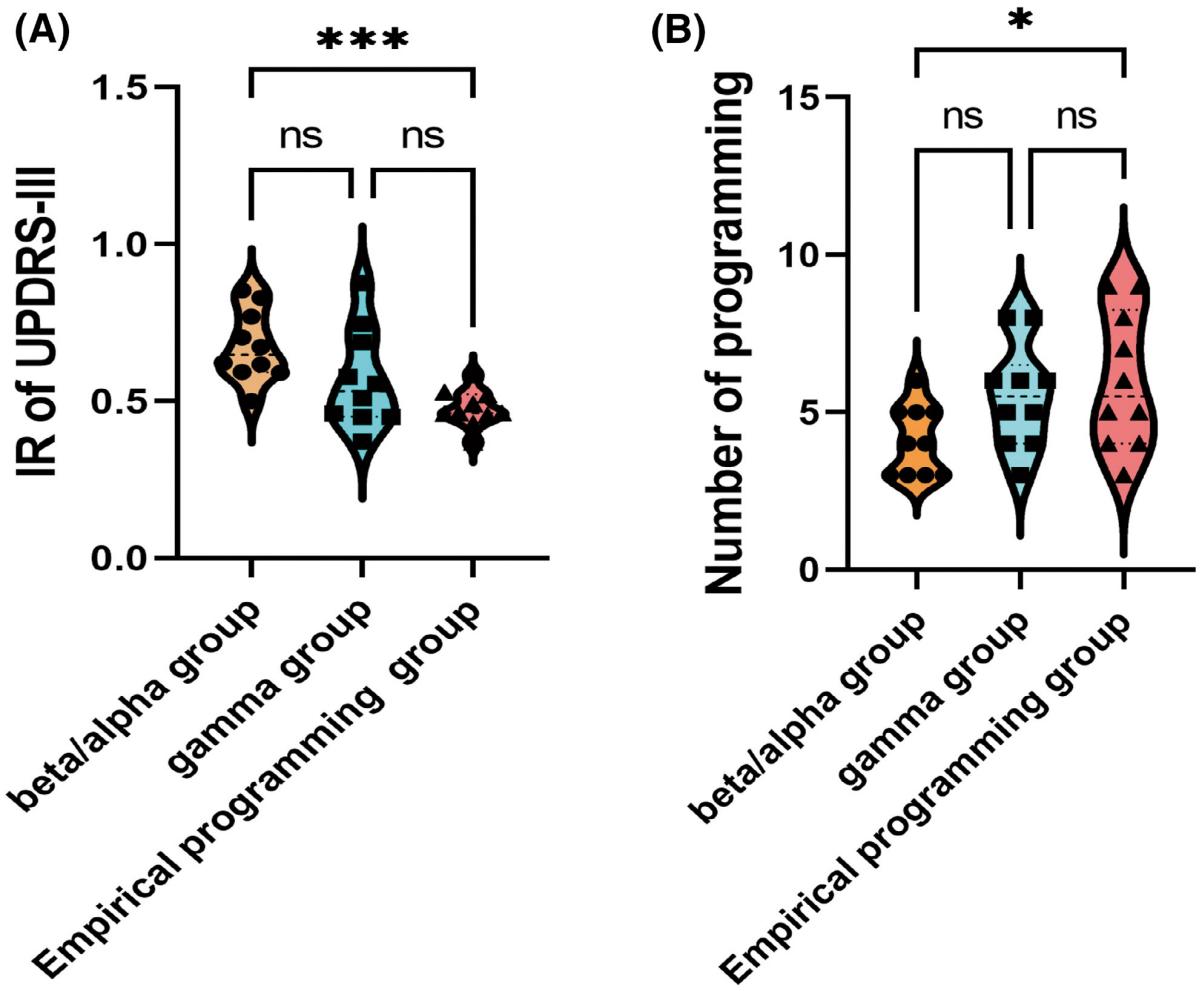
(A) Violin plots comparing the IR of UPDRS‐III between the three groups after 6 months of programming. The value for the beta/alpha group was significantly different from the empirical programming group. (B) Violin plots comparing the number of programmings between the three groups within 6 months after start‐up. As shown, the value for the beta/alpha group is different from that for the empirical programming group. *p < 0.05; ***p < 0.001.

The PVG group was subdivided into three groups for comparison: beta/alpha, gamma, and empirical programming groups. As shown in Table 2, after 6 months of programming, there were significant differences in the IR of UPDRS‐III and the number of programmings between the beta/alpha and empirical programming groups. However, no statistically significant changes could be observed for post‐LED, IR‐PDQ39, or the contact change rate. Furthermore, there were no statistically significant differences in IR for UPDRS‐III, post‐LED, IR‐PDQ39, number of programmings, or the contact change rate between the gamma and empirical programming groups. As shown by the violin plots in Figure 4A,B, the IR of UPDRS‐III and the programmings for the beta/alpha group were significantly higher than the empirical programming group.

**TABLE 2:** Clinical characteristics of patients in different groups after 6 months of programming.

	Beta/alpha group *n* = 10	Gamma group *n* = 10	Empirical programming group *n* = 10
IR of UPDRS‐III	0.68 ± 0.11***	0.57 ± 0.16	0.48 ± 0.06
Post‐LED (mg)	295.00 ± 101.9	271.70 ± 47.82	258.35 (218.75,300)
IR‐ PDQ39	0.724 (0.188,0.841)	0.329 ± 0.336	0.393 ± 0.325
Number of programmings	4.1 ± 1.10*	5.5 ± 1.65	6 ± 2.16
Contact change rate	0.267 (0,0.625)	0.163 (0,0.25)	0.238 ± 0.148

*Note*: Data are presented as mean ± SD or median (25th–75th percentile).

Abbreviations: IR of UPDRS‐III, Unified Parkinson's Disease Rating Scale part III improvement rate during “med ‐off”; IR‐PDQ39, improvement rate of PDQ39; post‐LED, postoperative daily levodopa equivalent dose.

* *p* < 0.05 vs. empirical programming group,

*** *p* < 0.001 vs. empirical programming group.Comparison among the gamma, beta/alpha, and empirical programming groups. We first confirmed whether the data of each group met a normal distribution and then performed a test of homogeneity of variance. The methods used for group comparisons were determined based on the results of these tests.

## 4 DISCUSSION

The neural function of the brain depends on the bioelectrical activity of neurons at all levels, generating specific potential changes. LFP indicates the sum of excitatory and inhibitory postsynaptic potentials responding to the local neuronal network. This highlights the synergistic effect of each neuron inside the neural network. It is well established that the leading cause of PD is degeneration and necrosis of dopaminergic neurons in the substantia nigra.^23^ When the number of dopaminergic neurons in this region is reduced, the produced dopamine cannot meet normal neural signal transmission needs, and PD symptoms will occur.^24^ Although drugs and DBS surgery can improve motor symptoms, currently, there is no means to cure or delay PD progression. The primary mechanism of PD drug action is to supplement dopamine or increase the sensitivity of dopamine receptors. However, the specific mechanism of DBS action remains unclear.^11^ DBS activates neuronal calcium and sodium channels, thereby affecting the release of neurotransmitters and transmission of electrical signals.^25^, ^26^ As the STN is part of the indirect signaling pathway of the basal ganglia, the primary effect of STN‐DBS may be to inhibit the movement of non‐active muscle groups. DBS is an effective and reliable way to obtain deep brain electrical signals. The value of studying the mechanism of DBS by analyzing deep brain electrical signals is increasingly recognized. Stimulating contacts with DBS electrodes changes the electrical signals of the surrounding clusters of neurons, which are LFP signals. Through LFP analysis, this study observed that DBS can change LFP signals around the stimulating contacts. The local field potential signals are categorized into different frequency bands, including delta, theta, alpha, beta, gamma, etc., from the frequency domain. Each frequency band signal has a different physiological significance, primarily associated with the state of activity level of the central nervous system. Specifically, the results indicate that DBS can change gamma oscillations and the beta/alpha oscillation ratios in the field potential signal and could reduce the PSD of gamma oscillations and the beta/alpha oscillation ratios. We speculate that alpha, beta, and gamma oscillations are associated with the indirect pathway of electrical signaling between the subthalamic nucleus and the medial nucleus of the pallidum and the substantia nigra pars reticulata. Therefore, DBS inhibits the excitatory transmitter glutamate release from neurons by reducing the oscillation energies. Thus, gamma oscillations and the beta/alpha oscillations ratio should be considered LFP biomarkers for further programming validation. We validated our results and identified that the beta/alpha oscillation ratios were superior for guiding programming. The IR of UPDRS III in the beta/alpha ratio group was significantly higher than in the traditional empirical programming group. Moreover, the number of programmings in this group was lowest within 6 months after the IPG switch‐on. No significant differences were observed between the gamma oscillation and empirical groups regarding IR of UPDRS‐III, post‐LED, IR‐ PDQ39, number of programmings, and contact change rate. These findings suggest that the beta/alpha oscillations ratio and gamma oscillations could replace empirical programming in postoperative programming. Although beta oscillations have been associated with symptoms in previous studies,^16^, ^27^ the present study did not obtain a positive result. This inconsistency may relate to the amount of data, failure to subclassify beta oscillations into low and high beta,^28^ or the different symptom subtypes of the enrolled patients. However, some studies have reported that beta oscillations are not associated with PD severity and that the beta oscillation energy value increases in different diseases. Due to ethical issues, collecting deep brain field potentials in healthy people is impossible. Thus, whether the beta oscillation energy value also increases in the deep brain of normal people and the value of beta oscillations as a marker should be further verified.^21^, ^29^

Based on the overall findings of this study, we make the following inferences. The difficulty and the time spent programming will increase with the popularity of directional electrodes and more stimulating contact appearances. Using field potential biomarkers can shorten the programming time, improve programming efficiency, and allow inexperienced doctors to perform neuromodulation operations. At our center, preoperative imaging is usually used for localization, and single cell spike potential signals are recorded during surgery to determine the electrode implantation position. Our findings suggest that field potential markers can be used instead of single‐cell electrical signals to determine the intraoperative electrode implantation position. In addition, with recent technological progress, LFP biomarkers are equally valuable for developing adaptive DBS. Given that DBS can change deep brain LFP signals to improve symptoms in PD, studies of LFP in dystonia, essential tremors, and other movement disorders may also be significant.

One limitation of this study is the sample size and limited follow‐up time. Long‐term clinical data for more patients are needed to confirm the findings. In this study, patients were divided according to the conventional oscillation interval. Future studies should optimize the oscillation interval range. Analysis of other potential biomarkers (blood, imaging, anatomical, etc.) may be more meaningful for overall PD management.

## 5 CONCLUSION

Overall, the findings of this study support that DBS can reduce the gamma oscillation signal energy of LFPs around the electrode contacts and the beta/alpha oscillation energy ratio. Gamma oscillations and the beta/alpha oscillation ratios may be neuroregulatory biomarkers for postoperative programming. The beta/alpha oscillation energy ratio may better guide empirical programming. LFP biomarkers could guide the depth of electrode implantation during surgery from the perspective of LFP instead of single‐cell electrical signals.

## CONFLICT OF INTEREST STATEMENT

The authors have no competing financial interests.

## CONSENT FOR PUBLICATION

All the authors have approved the final version of the manuscript for publication.

## Data Availability

The data that support the findings of this study are available on request from the corresponding author. The data are not publicly available due to privacy or ethical restrictions.
